# Associations Between Social Media Use and Mental Disorders in Adolescents and Young Adults: A Systematic Review and Meta-Analysis of Recent Evidence

**DOI:** 10.3390/bs15111450

**Published:** 2025-10-24

**Authors:** Hector Cabezas-Klinger, Fabian Felipe Fernandez-Daza, Yecid Mina-Paz

**Affiliations:** 1 Facultad de Ciencias Básicas, Grupo de investigación GIMIA, Universidad Santiago de Cali (Colombia), Cali 76001, Colombia; hector.cabezas02@usc.edu.co; 2 Facultad de Educación a Distancia y Virtual, Institución Universitaria Antonio José Camacho (Colombia), Cali 76001, Colombia

**Keywords:** risk factors, mental health, prevention, adolescents, youth, social networks, suicidal ideation

## Abstract

The exponential growth of human interactions on social media via the internet has revolutionized global communication, but it has also emerged as a critical factor in mental health linked to suicidal ideation and mental disorders. This systematic review and meta-analysis aimed to synthesize evidence on the most prevalent disorders in adolescents and young adults associated with social media use based on previous research, highlighting risk factors and key findings. Publications from 2020 to 2024 in highly relevant databases were reviewed following the PRISMA declaration guidelines. The meta-analysis (conducted in R software) of the included documents (24 studies, 68 effects) verified a significant and positive association between exposure to risk factors in social networks and various disorders in adolescents and young adults (aggregate correlation *r* = 0.2173, 95% CI [0.1826, 0.2520], *p* ≤ 0.0001), although with high heterogeneity (*I*^2^ = 99.66%). Prevention strategies were indicated by revealing data from contexts in which 40% of adolescents who died by suicide had developed online identities focused on suicidal thoughts.

## 1. Introduction

Global communication has been reshaped by the exponential growth of human interactions through social networking sites (SNS). However, SNS use has also emerged as a critical factor in mental health, particularly linked to suicidal ideation and attempts among adolescents a phenomenon with alarming global incidence (Szlyk et al., 2023; GBD 2021 Diseases and Injuries Collaborators, 2024). Although these platforms facilitate community health interventions (Shunmugam et al., 2023; Khosla et al., 2023), excessive use drives human psychology toward unprecedented risks: exposure to harmful content (e.g., self-harm narratives, cyberbullying) and the distortion of digital self-image are directly associated with suicidal crises, even more so than with other mental disorders such as eating disorders (Frieiro et al., 2022) or violent behaviors (Vannucci et al., 2020).

Among adolescents, pathological SNS use frequently tied to online video gaming (Tremolada et al., 2022; Moro et al., 2022) and associated with profiles of Internet Gaming Disorder (Labrador et al., 2023), not only threatens psychosocial development (Leibenluft & Barch, 2021), but also activates trajectories of suicide risk, particularly among those with early maturation (Kieling et al., 2024). Recent studies highlight that interactions on these platforms foster perceptions of isolation, hopelessness, and negative social comparison, mechanisms that account for their association with recurrent suicidal ideation (Halvorsen et al., 2021; Pop et al., 2022). Despite legislative initiatives to regulate SNS use among minors (Ministerio de Tecnologías de la Información y las Comunicaciones de Colombia, 2024; BBC News World, 2024, e.g., the recent law in Florida, USA), the paucity of research on the traceability of suicide risk-related posts and on mental disorders associated with SNS constrains effective preventive strategies.

While SNSs are useful tools for health promotion (Farsi, 2021; Farsi et al., 2022; Yang et al., 2022), their dual nature demands prioritizing an understanding of their role in the adolescent suicide crisis, a pressing problem that exceeds the burden of other psychopathologies (Wang et al., 2023). This dual role extends to other digital technologies, such as online games, which have been scrutinized for their complex impact on adolescent mental health during the COVID-19 pandemic (Gonzalez-Torres, 2024). Therefore, this study aims to address the following question: What associations exist between excessive or problematic SNS use in child and adolescent populations and the presence of DSM-5-specific mental disorders (American Psychiatric Association, 2022), such as depression or anxiety, and even suicidal behavior, compared with adolescents whose SNS use is moderate or low?

### Objective

This systematic review and meta-analysis aims to synthesize scientific evidence on the proposed mechanisms that account for the associations between excessive or problematic use of social networking sites (SNS) among children and adolescents and the presence of DSM-5–specific mental disorders, and to identify potential moderating factors that influence these relationships.

## 2. Methodology

To achieve this objective, the study employs a retrospective systematic review and meta-analysis, enabling theoretical integration through a review of highly relevant scientific literature in the field and the quantitative synthesis of results on a specific topic (Newman & Gough, 2020), while identifying conceptual categories and their potential associations.

The protocol for this review was preregistered in PROSPERO (registration number 1138492).

### Procedure

This systematic review and meta-analysis adhered to the Prefered Reporting Items for Systematic Reviews and Meta-Analyses (PRISMA) 2020 statement (Page et al., 2021). The databases Scopus, PubMed, Dimensions, and Lens were selected for their interdisciplinary relevance and technical coverage in the health sciences (Elsevier, 2019; Singh et al., 2020; Penfold, 2020; Paz-Pacheco, 2023). The search strategy employed Boolean operators—OR (to broaden results with related terms) and AND (to restrict them). Initially, the terms “risk factors” and “prevention” were linked with OR (in title, abstract, or keywords) and restricted using AND with “mental health” OR “mental disorder” OR “psychiatric disorders” OR “suicid” OR “self-harm.” Subsequently, social media terms (“social networks,” “social media,” etc.) were integrated with OR, and the query was constrained with AND to “adolescen” OR “youth” OR “young adult” OR “child*” to comprehensively capture the target population. The search was limited to English- and Spanish-language articles published between 2020 and 2024 to capture the most recent evidence following the onset of the COVID-19 pandemic, a critical period for SNS use and mental health (Table 1).

Across the four databases, 1668 records were identified for the period 2020–2024. Records meeting the predefined inclusion and exclusion criteria proceeded to data extraction (Table 2). Age-related inclusion criteria were clearly defined as adolescents (12–17 years, per WHO) and young adults (18–24 years) to capture the critical transition period. Studies that included only adults (>25 years) were excluded, unless they reported data disaggregated for the target age range.

**Figure 1:**
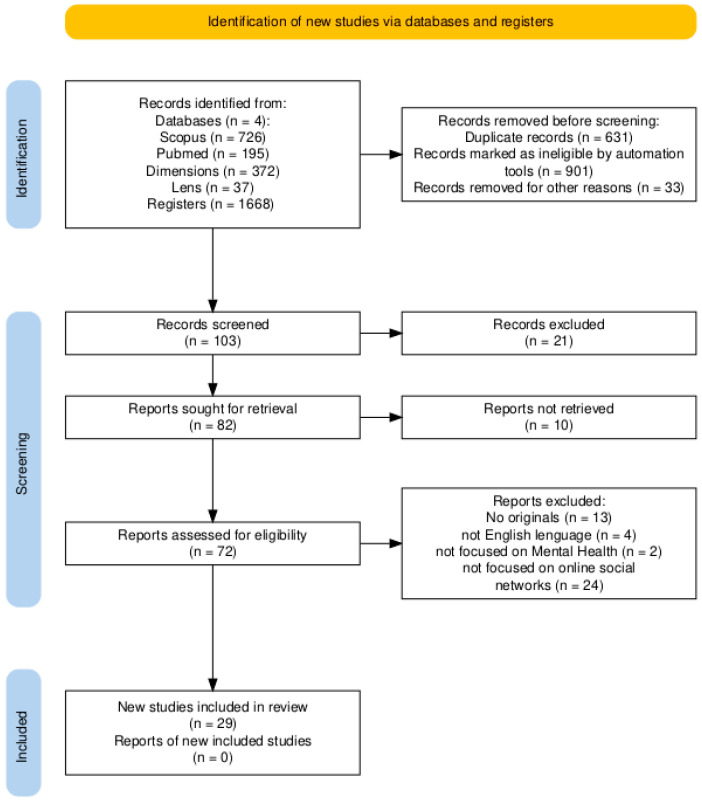
Study selection based on the PRISMA 2020 flow diagram. Figure created by the authors using (Haddaway et al., 2022).

A screening process was conducted to remove potential duplicate records and documents not relevant to the research line by reviewing titles, keywords, and abstracts. A log was maintained documenting the classification of the records that ultimately comprised the analytical sample. Figure 1 presents the flow diagram detailing the phases of the document selection process, updated to the PRISMA 2020 standard.

## 3. Results

### 3.1. Qualitative Synthesis

A synthesis of the characteristics and principal findings from the 29 included studies, organized by the main identified impact dimensions, is presented in Table 3. The complete data-extraction file, including methodological details, study populations, and specific findings, is available in Table S1 of the Supplementary Material. Likewise, the risk-of-bias assessment for each study is detailed in Table S2 of the Supplementary Material.

Based on the reading and analysis of all included documents, 72.4% focused exclusively on adolescents, whereas 17.2% focused exclusively on young adults. 93% were original research articles; the remainder comprised two case studies. Eight categories were identified as the principal mental-health problems related to SNS use. The literature spans a wide range of contexts in which these disorders are of scientific interest, from implications for neurodevelopment in minors (Ávalos Ruiz et al., 2022) and investigations conducted during COVID-19 lockdowns (Yıldırım et al., 2023) to threats to the well-being of medical students attributed to problematic SNS use (Sserunkuuma et al., 2023), as detailed below. To synthesize and organize the findings, identified mental disorders were categorized within the conceptual framework of internalizing and externalizing spectra, a robust, validated model that continues to demonstrate utility in contemporary psychopathology (Kotov et al., 2017; Conway et al., 2019). This dichotomy, foundational to dimensional models such as the Hierarchical Taxonomy of Psychopathology (HiTOP), groups conditions according to the dominant direction of symptom expression: inward toward the self (internalizing, e.g., depression, anxiety, suicidal ideation) versus outward toward the external environment (externalizing, e.g., aggression, impulsivity, rule-breaking). This classification is particularly useful for analyzing the impact of social media, as distinct digital dynamics (e.g., social comparison vs. cyberbullying) may differentially predispose individuals toward one spectrum or the other.

### 3.2. Internalizing Disorders

The literature documents significant correlations between SNS use and internalizing disorders, with clearly identified pathogenic mechanisms. Suicidal ideation and behavior. Szlyk et al. (2023) found that 65% of youth with suicidal ideation and 80% with non-suicidal self-injury (NSSI) perceived emotional triggers when posting depressive content. Abrutyn et al. (2020) showed that exposure to suicides on SNS can normalize the behavior, reframing academic pressures as plausible causes, while Rossi and DeSilva (2020) documented cases in which ambivalent comments on Snapchat precipitated suicide attempts. Balt et al. (2023) reported that 40% of adolescents who died by suicide had developed online identities centered on suicidal thoughts, amplified by algorithms that prioritize negative content. Depression and anxiety. Shensa et al. (2020) identified that digital social support can increase depressive risk due to a lack of emotional reciprocity, in contrast with the protective effect of face-to-face contact. Tajjamul and Aleem (2022) associated nighttime SNS use with sleep disturbances in 43.7% of Pakistani university students, and Agbo (2021) linked >10 h/day on WhatsApp with depressive symptoms among Nigerian adolescents (r = 0.42 with social comparison). Kreski et al. (2021) identified gender-differential effects: females at otherwise low risk exhibited greater susceptibility to depressive symptoms with daily SNS use. Problematic/”addictive” SNS use and comorbidity. Borraccino et al. (2022) reported problematic social media use (PSMU) in 8.8% of Italian adolescents, correlated with cybervictimization (OR = 2.10–2.43). Victor et al. (2024) found prevalences of 72.7% (addiction) and 38.8% (depression) among Malaysian adolescents, with higher risk among females exceeding 5 h/day of use. Sserunkuuma et al. (2023) corroborated this linkage among Ugandan medical students, mediated by academic stress. Associated conditions. Additional associations include metabolic alterations (Saintila et al., 2024: OR = 1.517–2.596 for metabolic syndrome in males >16 years), eating disorders (Farias et al., 2024: OR = 3.39–10.7 with exposure to diet influencers), and insomnia (Hamilton & Lee, 2021: β = 0.70–0.76 for daytime sleepiness; Yıldırım et al., 2023: mediation by fear of COVID-19, β = 0.32).

### 3.3. Externalizing Disorders

Externalizing disorders manifest disruptive behavioral patterns that are exacerbated by digital dynamics, with quantifiable dimensions of risk. Cyberbullying and digital aggression. Parris et al. (2022) showed that perceived school bullying increased rumination about digital interactions (β = 0.13, *p* < 0.05) and emotional stress (β = 0.37, *p* < 0.001), although anonymity in cyberbullying mitigated these effects. de Felice et al. (2022) identified, among Italian adolescents, risk behaviors such as pathological popularity-seeking (d = 0.78) and displays of violence, with gender dimorphism: females reported greater impact from unrealistic body models (Δ = 32% vs. males). Craig et al. (2020), across 42 countries, found that problematic social media use (PSMU) increased risk of cybervictimization (adjusted RR = 1.48; 95% CI: 1.42–1.55) and cyberbullying perpetration (RR = 1.84; 95% CI: 1.74–1.95), with significant associations for perpetration in 86% of countries. This effect was ~30% greater among females (*p* < 0.01), attributed to comparative exposure to body ideals. Borraccino et al. (2022) likewise found PSMU associated with cybervictimization (OR = 2.10–2.43) and perpetration (OR = 2.29–2.78), while emphasizing that family and school support reduced these risks by up to 56% (*p* < 0.01). Substance use. Oksanen et al. (2021) reported that 2% of adolescents acquired drugs via Instagram/Facebook, linked to low self-regulation (β = −0.41), psychological distress (β = 0.38), and concurrent addictive behaviors. In a network analysis approach, Martínez-Fernández et al. (2021) further illustrated that cannabis use among adolescents during COVID-19 confinement was associated with lower emotional intelligence and peer networks centered on substance use. Pravosud et al. (2024) associated increased SNS use during lockdown with e-cigarette use as a coping mechanism (OR = 4.06), mediated by loneliness (OR = 3.33). Complementing this, Varela et al. (2023) found that Chilean adolescents with higher social media addiction during the pandemic reported lower wellbeing and greater use of maladaptive coping strategies, which in turn exacerbated psychological distress. Zani et al. (2024) extended these findings, showing that 52.9% of Indonesian adolescents exposed to alcohol/drug content on SNS developed higher risk of use (r = 0.29), with associated academic deterioration (r = −0.241).

### 3.4. Emerging Patterns and Underlying Mechanisms

Qualitative analysis identified the following cross-cutting patterns and mechanisms:

- Normalization of maladaptive behaviors via repeated exposure to violent or suicide-related content (Abrutyn et al., 2020; Rossi & DeSilva, 2020).
- Behavioral disinhibition facilitated by anonymity in interactions with strangers ((Craig et al., 2020): RR = 1.40 for cyberbullying in 28% of countries).
- Maladaptive stress management through substance use (Pravosud et al., 2024) or compulsive SNS use (Victor et al., 2024).
- Diffusion of aggressive or unrealistic models in digital communities, exacerbating negative social comparison and body dissatisfaction (de Felice et al., 2022; Farias et al., 2024).
- Disruption of sleep–wake cycles due to nocturnal use and hyperconnectivity, impacting mood and emotion regulation (Tajjamul & Aleem, 2022; Hamilton & Lee, 2021).

These mechanisms operate interactively and are often moderated by individual factors (e.g., gender, prior vulnerability) and contextual factors (e.g., cultural norms, social support).

Critical Appraisal (Methodological Quality and Risk of Bias)

Study designs. Cross-sectional studies constituted 79% of the included evidence.

Appraisal tools.

- AXIS (20 items; quantitative): Each item rated yes/no/partially; “yes” responses were tallied to determine compliance (Downes et al., 2016).
- CASPe (10 items; qualitative) (CASPe, 2020): Items scored 0–2; studies scoring ≥18 points (90%) were considered high quality.
- Tools were applied jointly alongside STROBE and ROBINS-I for exposure/transversal designs (von Elm et al., 2007; Vandenbroucke et al., 2014; Sterne et al., 2016).

Methodological quality. Overall strengths included transparency (88% compliance), use of validated scales (75%), confounder control, ethics (95%), and funding statements. The overall risk of bias was moderate, primarily due to non-probabilistic sampling (78%), absence of power calculations (65%), omission of response rates (82%), handling of missing data, and reliance on self-reports (25%, desirability bias). See Figure S1 (Methodological quality and risk of bias, Supplementary Material).

### 3.5. Meta-Analysis

Of the 29 studies registered for analysis, 24 reported effect sizes using diverse metrics; these were transformed to Pearson correlations (r) as a common metric. A meta-regression was conducted to estimate the pooled effect while accounting for heterogeneity in study design, sample sizes, countries, and age group (see Table S3, Supplementary Material) (Viechtbauer & López-López, 2022; López-López et al., 2014). The remaining studies were synthesized qualitatively.

**Figure 2:**
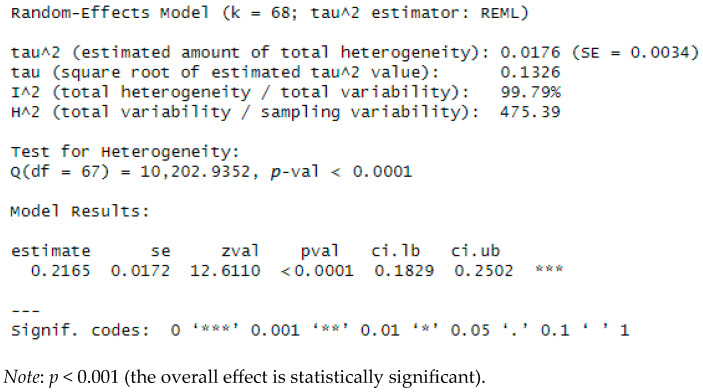
Pooled effect (meta-analysis). Effect size (Fisher’s *z*-transformed correlation) = 0.2165 (95% CI: 0.1829–0.2502).

A random-effects meta-analysis (performed in R version 4.5.0 (R Core Team, 2025) using the *metafor* package) examined the overall association between exposure to SNS risk factors among adolescents/young adults and mental disorders (including suicidal behavior), across 68 outcomes from 24 studies (some studies reported multiple effects, e.g., ef1, ef2; see Table S3). Results indicated a significant positive association between the two factors (Z = 0.2165, SE = 0.0172, 95% CI [0.1829, 0.2502]; Z = 12.6110, *p* ≤ 0.0001). However, substantial heterogeneity was observed in effect sizes among included studies (τ2 = 0.0176, SE = 0.0034; τ = 0.1326; Q(67) = 10,202.9352, *p* < 0.0001; I^2^ = 99.79%). Accordingly, results should be interpreted with caution, given the high between-study variability (see Figure 2).

The foregoing results indicate substantial between-study variability, as the Q test was significant (Q(67), *p* < 0.0001). The I^2^ estimate (99.79%) suggests that nearly all observed variance reflects differences in effect sizes rather than sampling error. The pooled association, expressed as Fisher’s z = 0.2165, corresponds to Pearson’s r = 0.2132, representing the combined effect between the mental disorders reported across studies and overall social media use. This pooled effect differed significantly from zero (z = 12.6110, *p* < 0.0001).

**Figure 3:**
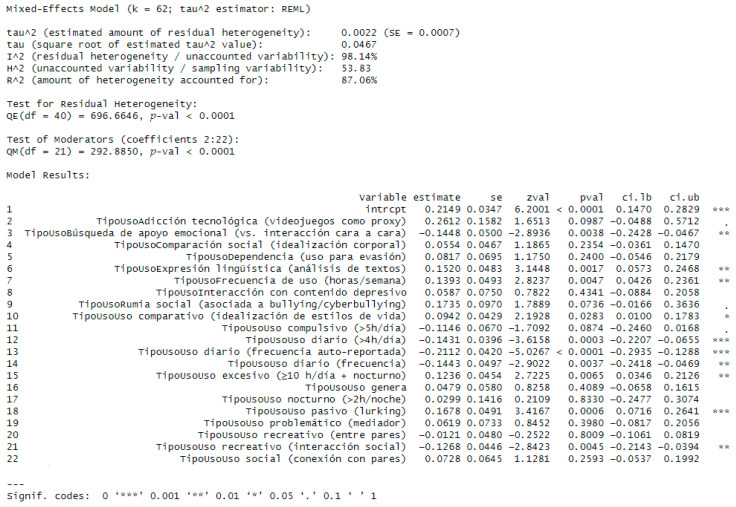
Meta-regression: Explained variability (R^2^ > 87%).

Influence diagnostics were conducted in three phases (see Figure S2, Supplementary Material). Six effect sizes were flagged for exclusion—two from (Hamilton & Lee, 2021), two from (Sserunkuuma et al., 2023), and two from (Nilsen et al., 2023)—to reduce potential bias in the pooled estimate (Cooper et al., 2019). Given the variability and heterogeneity, a random-effects meta-regression was performed to confirm the overall association and to explore sources of heterogeneity (see Figure 3). The model included type of social media use (UseType) as a moderator, with category values detailed in Table S3 (Supplementary Material).

The meta-regression results display the categories of the TypeUse moderator. The intercept (intrcpt = 0.2149) remains close to the pooled effect estimated previously in the meta-analysis. Inclusion of Type Use explained >80% of the between-study variability (R^2^ = 87.06%). Despite the limitations noted, the findings suggest that exposure of adolescents and young adults to social media risk factors is associated with adverse mental-health outcomes. In aggregate, the results do not show null effects across the different types of social media use among adolescents (see Figure S1, Supplementary Material).

**Figure 4:**
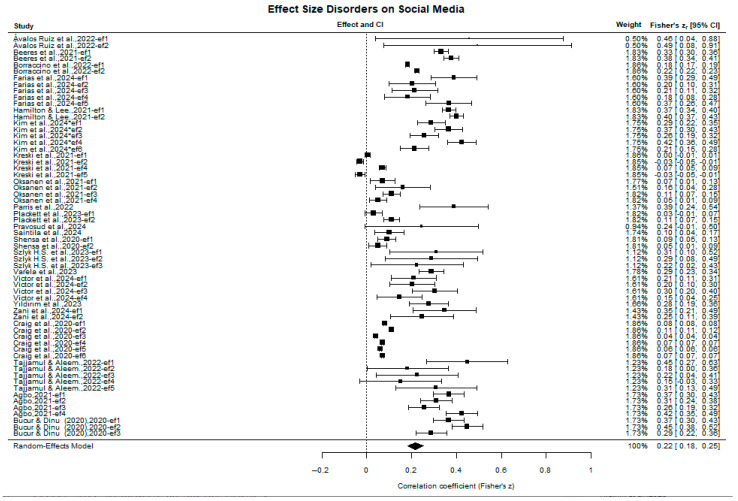
Forest plot of individual effect sizes and pooled effect (Fisher’s z), with 95% confidence intervals. The included studies are: (Ávalos Ruiz et al., 2022; Beeres et al., 2021; Borraccino et al., 2022; Farias et al., 2024; Hamilton & Lee, 2021; Kim et al., 2024; Kreski et al., 2021; Oksanen et al., 2021; Parris et al., 2022; Plackett et al., 2023; Pravosud et al., 2024; Saintila et al., 2024; Shensa et al., 2020; Szlyk et al., 2023; Varela et al., 2023; Victor et al., 2024; Yıldırım et al., 2023; Zani et al., 2024; Craig et al., 2020; Tajjamul & Aleem, 2022; Agbo, 2021; Bucur & Dinu, 2020).

Figure 4 (forest plot) presents the individual studies with their effect-size estimates and visually highlights the high heterogeneity across studies. Even so, the pooled effect size trends toward r ≈ 0.22; because the pooled-effect diamond does not cross the zero-effect line, the combined effect is statistically significant (see Fisher’s z diamond at the bottom of Figure 4). This pattern indicates that problematic social media use is significantly associated with a higher incidence of mental-health disorders in child and adolescent populations, functioning as a potential risk factor in observational studies.

### 3.6. Publication Bias Risk Assessment

**Figure 5:**
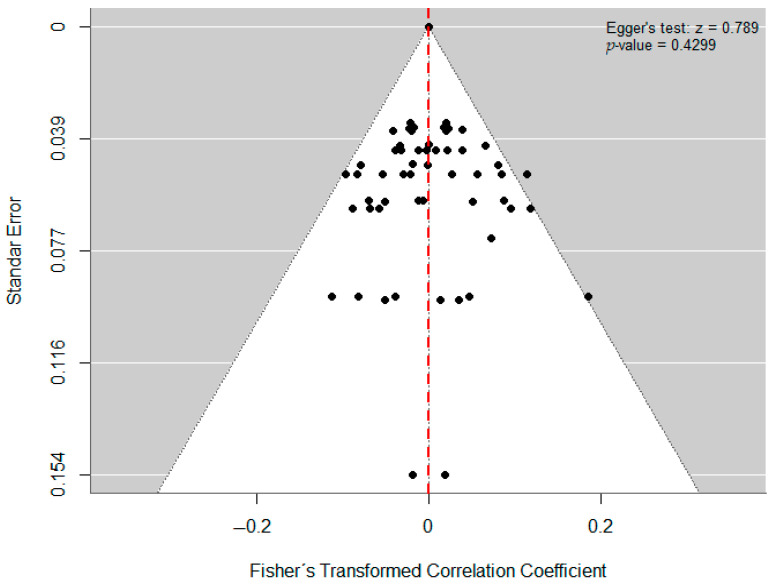
Funnel plot.

**Figure 6:**
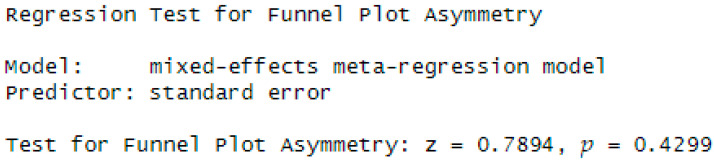
Test de Egger.

We assessed publication-bias risk using Egger’s regression test and the funnel plot (Lin & Chu, 2018; Egger et al., 1997) (Figure 5 and Figure 6). We tested the following:

- H_0_: no publication bias (intercept = 0);
- H_1_: possible publication bias (intercept ≠ 0); reject H_0_ if *p* < 0.05.

Visual inspection of the model’s funnel plot (Figure 5) indicates a symmetric, well-shaped distribution, with no obvious small-study effects. There is no clear visual evidence that publication bias inflated the pooled effect size, suggesting that the included evidence is a reasonable representation of the existing literature on the topic.

Egger’s regression test yielded *p* = 0.4299 > 0.05 and z = 0.7894, indicating no statistical evidence to reject the null hypothesis of a zero intercept (no publication bias). The small *z* statistic—close to zero—suggests no substantial deviation from funnel-plot symmetry. Collectively, these results imply that the model adequately captures precision (i.e., no discernible small-study effects), and there is no evidence of publication bias or significant asymmetry in the funnel plot.

## 4. Discussion

This systematic review and meta-analysis synthesized recent evidence (2020–2024) on the association between social networking site (SNS) use and mental disorders, including suicidal ideation and behavior among adolescents and young adults. The meta-analysis of 24 quantitative studies revealed a significant positive association, small to moderate in magnitude (Pearson’s correlation r = 0.2165), between exposure to SNS-related risk factors and mental health problems, consistent with prior reviews (Valkenburg et al., 2022; Alonzo et al., 2021). However, the very high heterogeneity (I^2^ = 99.79%) underscores the complexity of this relationship and the critical role of moderating factors (e.g., UseType, the type of SNS use, which accounted for variability in the meta-regression). Our qualitative findings deepen understanding of the mechanisms and contexts that explain this heterogeneity. The association appears to be mediated by multiple pathways, including negative social comparison (Agbo, 2021; de Felice et al., 2022), cyberbullying (Craig et al., 2020; Borraccino et al., 2022), sleep disruption (Tajjamul & Aleem, 2022; Hamilton & Lee, 2021), and the normalization of risk behaviors such as suicide (Abrutyn et al., 2020; Balt et al., 2023) or substance use (Oksanen et al., 2021; Pravosud et al., 2024). It is crucial to note the role of algorithms in creating echo chambers that amplify and reinforce negative content (Balt et al., 2023), generating unique risk trajectories for vulnerable users. Marked cultural and geographic differences (e.g., Kim et al., 2024 in South Korea; Agbo, 2021 in Nigeria; Saintila et al., 2024 in Peru) indicate that the impact of SNS is not universal. Furthermore, the relationship differs across developmental stages, as evidenced by distinct patterns of association between SNS use and psychosocial well-being in older adults (Ho et al., 2023). Factors such as mental-health stigma, social norms around success, access to psychosocial support, and the level of critical digital literacy substantially modulate the relationship. For example, the lack of correlation found in some settings (Tajjamul & Aleem, 2022) may reflect normalization of use without awareness of adverse effects or unmeasured cultural protective factors. Despite the robustness of the association, the predominance of cross-sectional designs (79%) limits causal inference. Pre-existing vulnerabilities may predispose individuals both to problematic SNS use and to mental health problems (selection hypothesis), or the influence may be bidirectional. The few longitudinal studies included (Beeres et al., 2021; Plackett et al., 2023) point to complex bidirectional effects, whereby SNS use can exacerbate symptoms, while symptoms can also increase use as a maladaptive coping strategy. Future research should prioritize longitudinal and experimental designs to disentangle causal relations and identify high-risk subgroups.

Limitations of this review include the exclusion of gray literature (subtly suggested in the meta-regression) and potential publication bias, although statistical tests did not detect it. The high heterogeneity explored via meta-regression suggests additional unmeasured factors (e.g., specific platform type, personality traits) that influence outcomes. The reliance on self-report measures introduces further potential bias. Nevertheless, this review offers an updated, comprehensive quantitative and qualitative synthesis of the state of the art.

## 5. Conclusions

Despite the limitations imposed on cross-sectional design studies regarding their level of evidence, the results show the high methodological quality of the documentary corpus and a moderately low level of risk. Exposure to risk factors on social media (SMP) exerts a heterogeneous yet profoundly harmful impact on adolescents and young adults, mediated by cultural contexts and unique psychosocial dynamics. In South Korea, Kim et al. (2024) revealed that female adolescents exposed to online sexual victimization (e.g., unwanted solicitations or harassment) had a 2.3 times higher risk of suicidal ideation, especially if they had a history of childhood adversities (ACEs). This finding is exacerbated by the normalization of academic and work success standards on platforms like Instagram, where social comparison intensifies body dissatisfaction and isolation (de Felice et al., 2022). Meanwhile, in Nigeria, Agbo (2021) identified that young people who spent more than 10 h per week on SMP showed depression rates 40% higher than the average, linked to the internalization of school failure and pressure to emulate unrealistic lifestyles. In contrast, in Pakistan, Tajjamul and Aleem (2022) found no significant correlation, suggesting that prolonged exposure to SMP might be normalized without awareness of its effects, a phenomenon that de Felice et al. (2022) attribute to the lack of critical digital literacy in certain environments. However, in cohesive communities like Poplar Grove (U.S.), Abrutyn et al. (2020) demonstrated that the dissemination of local narratives on SMP (e.g., attributing suicides to social pressure) reconfigures youth perceptions, making the act “imaginable” even in the absence of mental disorders. Together, these studies (based on mixed, qualitative, and quantitative methodologies) emphasize that SMP not only expose youth to immediate risks (cyberbullying, social comparison) but also reproduce structural inequalities: from the glorification of unattainable models in competitive societies to the invisibility of mental disorders in cultures with deeply rooted stigmas. The evidence calls for interventions that, rather than restricting access, empower adolescents to navigate these spaces with culturally informed critique and real support networks. All of the above, along with the combined effect from the studies that reported statistical results, suggest that various mental health disorders in adolescents and young adults are significantly associated with exposure to risk factors on SMP.

### Recommendations

Research.

1. Conduct longitudinal and intervention studies to establish causality and evaluate preventive strategies.
2. Investigate the platform- and algorithm-specific roles in risk amplification.
3. Examine protective factors (resilience, critical digital literacy, family/school support) across cultural contexts.
4. Employ mixed-methods approaches to capture the complexity of subjective experience.

Policy and Regulation.

1. Develop and enforce legal frameworks that protect minors’ privacy and well-being online (e.g., laws similar to those in Florida and Colombia).
2. Require greater algorithmic transparency from SNS companies and effective mechanisms for removing content promoting self-harm, suicide, cyberbullying, or unrealistic body ideals.
3. Integrate digital and mental health education into school curricula.

Clinical and Preventive Practice.

1. Implement early screening programs for problematic SNS use and comorbidities in primary care and school settings.
2. Develop psychoeducational interventions for adolescents and families that encourage critical, healthy SNS use, promote emotion regulation skills, and strengthen face-to-face interactions.
3. Create and promote accessible crisis-support resources via digital channels for at-risk youth.

## Figures and Tables

**Table 1:** Search expressions used in the consulted databases.

Database	Search Strategy
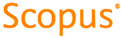	(TITLE-ABS-KEY (“risk factors” OR “prevention”) AND TITLE-ABS-KEY (“mental health” OR “mental disorder*”) AND TITLE-ABS-KEY (“Social Media” OR “Social Network*” OR “online social network*”) AND TITLE-ABS-KEY (“adolescents” OR “young adult”)) AND PUBYEAR > 2019 AND PUBYEAR < 2025 AND (LIMIT-TO (LANGUAGE, “English”))
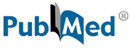	((“risk factor*”[Title/Abstract] OR “prevention”[Title/Abstract]) AND (“Mental Health”[Title/Abstract] OR “mental disorder*”[Title/Abstract]) AND (“social network*”[Title/Abstract] OR “Social Media”[Title/Abstract] OR “online social network*”[Title/Abstract]) AND (“adolescent*”[Title/Abstract] OR “young adult*”[Title/Abstract])) OR ((“Risk Factors”[MeSH Terms] OR “prevention and control”[MeSH Subheading]) AND (“Mental Health”[MeSH Terms] OR “Mental Disorders”[MeSH Terms]) AND (“Social Networking”[MeSH Terms] OR “Social Media”[MeSH Terms] OR “Online Social Networking”[MeSH Terms]) AND (“Adolescent”[MeSH Terms] OR “Young Adult”[MeSH Terms]))
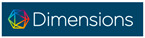	‘((“risk factor*”OR “prevention”) AND (“mental health” OR “mental disorder*”) AND (“social network*” OR “social media” OR “online social network*”) AND (“adolescent*” OR “young adult*”))’ in title and abstract, Publication Year is 2024 or 2023 or 2022 or 2021 or 2020
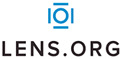	(title:(“risk factor*” OR “prevention”) OR abstract:(“risk factor*” OR “prevention”) OR keyword:(“risk factor*” OR “prevention”) OR field_of_study:(“risk factor*” OR “prevention”)) AND (title:(“mental health” OR “mental disorder*”) OR abstract:(“mental health” OR “mental disorder*”) OR keyword:(“mental health” OR “mental disorder*”) OR field_of_study:(“mental health” OR “mental disorder*”)) AND (title:(“social network*” OR “social media*”) OR abstract:(“social network*” OR “social media*”) OR keyword:(“social network*” OR “social media*”) OR field_of_study:(“social network*” OR “social media*”)) AND (title:(“adolescent*” OR “young adult*”) OR abstract:(“adolescent*” OR “young adult*”) OR keyword:(“adolescent*” OR “young adult*”) OR field_of_study:(“adolescent*” OR “young adult*”))

*Note*. The asterisk (*) is a truncation wildcard used in the search queries to include multiple word endings. For example, searching for disorder retrieves records containing “disorder” and “disorders”, and adolescen* retrieves “adolescent”, “adolescents”, and “adolescence”.

**Table 2:** Inclusion and exclusion criteria for the review.

Inclusion Criteria	Exclusion Criteria
Original article or case study on social media and mental health	Non-original article or not a case study on social media and mental health
Focused on social media	Not focused on social media
Documented mental health risks	Undocumented mental health risks
Proposals for prevention of mental health issues	No proposals for prevention of mental health issues
Applied to adolescents and young adults	Applied to another age group
Full access to the publication	No access to the full publication
Written in English	Written in another language

**Table 3:** Synthesis of impact dimensions, associated mechanisms, and findings.

Impact Dimension	Main Associated Mechanism	Key Finding Example (Reference Study)
Mental Health	Problematic use, social comparison, rumination	Greater social media use associated with more mental health symptoms (Beeres et al., 2021).
Suicidal Behavior	Normalization, dissemination, imitation of graphic content	Reinterpretation of suicide as a result of social pressure, not just mental illness (Abrutyn et al., 2020).
Cyberbullying	Problematic use as a key predictor of victimization and perpetration	Problematic use is the strongest predictor of cyberbullying in 42 countries (Craig et al., 2020).
Sleep Disturbances	Nighttime use, frequency of checking, displacement of rest time	Higher frequency of posting and checking social media associated with greater daytime sleepiness (Hamilton & Lee, 2021).
Body Image	Exposure to ideal models, influencers, and food advertising	Being female, preferring Twitter, and following food influencers associated with risk of eating disorders (Farias et al., 2024).
Substance Use	Exposure to content and access to providers through platforms	2% of youths purchased drugs online, primarily through Instagram/Facebook (Oksanen et al., 2021).
General Well-being	Addiction, substitution of face-to-face interactions, lower quality emotional support	Emotional support on social media was associated with higher risk of depression, face-to-face with lower risk (Shensa et al., 2020).

## Data Availability

All data supporting the findings of this study are contained within the article.

## Supplementary Materials

The following supporting information can be downloaded at: https://www.mdpi.com/article/10.3390/bs15111450/s1, Figure S1: Meta-regression: Explained variability (R2 > 87%) and effects on TypeUse categories; Figure S2: Diagnosis of influence on combined effect; Table S1: Data extraction; Table S2: Risk of bias of the included studies; Table S3: Effect sizes.
