# Supplementary material for: Associations Between Social Media Use and Mental Disorders in Adolescents and Young Adults: A Systematic Review and Meta-Analysis of Recent Evidence
Source: Behav Sci (Basel). 2025 Oct 24;15(11):1450. doi: 10.3390/bs15111450 (PMC12649677; doi:10.3390/bs15111450)
Supplement: Supplementary file 1 [file behavsci-15-01450-s001.zip › behavsci-3714940-supplementary.pdf]

Supplementary material

Table S1. Data extraction

Author(s)	Country	Findings	Title	Design	Objective	Population	Sample
Abrutyn et al. (2020)	USA	In the community studied (Poplar Grove), suicide was no longer attributed primarily to mental illness (the dominant narrative in the US) and was reinterpreted as a result of social pressure (academic demands, family, success).	Rekeying Cultural Scripts for Youth Suicide: How Social Networks Facilitate Suicide Diffusion and Suicide Clusters Following Exposure to Suicide	Qualitative (case study)	To analyze how social media facilitates the spread of suicide in communities.	Poplar Grove Community, USA	118 participantes
Agbo (2021)	Nigeria	They prefer to chat on WhatsApp/Facebook instead of talking to their parents (Table 1, item 2). 75% of the items assessed show excessive use (e.g., staying up until 2:00 AM, eating while chatting).	Social Media Use: A Risk Factor for Depression among School Adolescents in Enugu State of Nigeria	Descriptive cross-sectional study	To assess social media use as a risk factor for depression.	Schoolchildren in Enugu, Nigeria.	800 adolescentes.
Ávalos Ruiz et al. (2022)	Spain	Significant correlation between video game addiction and depression (r = 0.428; *p* = 0.033). Significant correlation between overall technology addiction (ADITEC) and bullying (r = 0.458; *p* = 0.021).	Use of ICT and social networks in the neurodevelopment of minors. Implications for the prevention of the risk of social exclusion	Observational cross-sectional study	To explore the relationship between technology addiction and risk factors for social exclusion.	Spanish adolescents (12–17 years old).	25 participantes.
Ballesteros-Holmes et al. (2024)	USA	Daily time on social media decreased from a median of 4.5 hours at baseline to 3 hours at 12 weeks	Decreasing Social Media Use Through Motivational Interviewing: A Pediatric Primary Care Quality Improvement Project	Pre-experimental (pre-post without control group)	To evaluate the effectiveness of an intervention based on motivational interviews and family media use plans to reduce social media use in adolescents with anxiety or depression, in a pediatric primary care setting.	Adolescents, 12–18	12
Balt et al. (2023)	Netherlands	Imitation of suicidal behavior: 13 adolescents (12 female) accessed graphic self-harm/suicide content on forums and social media.	Social media use of adolescents who died by suicide: lessons from a psychological autopsy study	Qualitative psychological autopsy study	To explore the role of social media in adolescent suicide.	Dutch adolescents who died by suicide.	35 casos.
Beeres et al. (2021)	Sweden	Adolescents with the greatest use of social media reported more symptoms of mental health problems	Social Media and Mental Health Among Early Adolescents in Sweden: A Longitudinal Study With 2-Year Follow-Up (KUPOL Study)	Longitudinal	To analyze the longitudinal relationship between social media use and mental health.	Swedish adolescents aged 14-15	3,501 participantes iniciales. 51,5% eran niñas.
Borraccino et al. (2022)	Italy	Problematic social media use is a significant risk factor for cybervictimization and perpetration.	Problematic Social Media Use and Cyber Aggression in Italian Adolescents: The Remarkable Role of Social Support	Cross-sectional	To analyze the relationship between problematic social media use, social support, and cyberbullying.	Italian adolescents aged 11-15	58,976 participantes
Bucur & Dinu (2020)	Romania	Depressed users: They write about negative feelings and stressful life events (e.g., "someone killed, lonely, I'm sad").	Detecting early onset of depression from social media text using learned confidence scores	Analytical cross-sectional study	To detect early signs of depression in Reddit texts using topic modeling and confidence scores.	Reddit users with mental health-related posts	820
Craig et al. (2020)	47 countries and regions in Europe and Canada	Problematic SMU is the strongest and most consistent predictor of cyberbullying (victimization and perpetration) in most countries.	Social Media Use and Cyber-Bullying: A Cross-National Analysis of Young People in 42 Countries	Cross-sectional observational study (based on data from the 2017-2018 Health Behavior in School-aged Children survey).	To analyze the association between types of social media use (problematic, intense, contact with strangers) and risk of cybervictimization/perpetration in adolescents.	Adolescents (11–15 years old)	Total: 180,919 participantes (muestra ponderada). Submuestra analítica: 166,647 participantes.

Author(s)	Country	Findings	Title	Design	Objective	Population	Sample
de Felice et al. (2022)	Italy	Constant exposure to an 'ideal role model,' perceived as very distant from their current situation, generates a very pronounced feeling of frustration, especially in girls.	How Do Adolescents Use Social Networks and What Are Their Potential Dangers? A Qualitative Study of Gender Differences	Qualitative (focus groups)	To explore gender differences in social media use and its perceived risks in adolescents.	Italian adolescents aged 13-18 in schools	296 participantes (166 mujeres, 130 hombres)
Farias et al. (2024)	Chile	Variables significantly associated with the risk of eating disorders: Being female; Preference for Twitter; Interest in food influencers; Frequent exposure to food advertising	Social networks and risk of eating disorders in Chilean young adults	Cross-sectional observational study	To analyze the relationship between social media use and risk of eating disorders.	Chilean young adults (20–35 years old).	370 participantes.
Hamilton & Lee (2021)	USA	Higher frequency of posting (B = 0.70, *p* < .001) and checking (B = 0.76, *p* < .001) social media is associated with greater daytime sleepiness.	Associations Between Social Media, Bedtime Technology Use Rules, and Daytime Sleepiness Among Adolescents: Cross-sectional Findings From a Nationally Representative Sample	Cross	To examine associations between social media use, parental rules, and daytime sleepiness in adolescents.	Adolescents aged 12-17 in the US	4,153 participantes
Kim et al. (2024)	South Korea	Adult women: Risk associated with mental disorders. Adolescents: Risk linked to interpersonal experiences	Understanding and comparing risk factors and subtypes in South Korean adult and adolescent women's suicidal ideation or suicide attempt using survey and social media data	Analytical cross-sectional study	To compare risk factors for suicidal ideation/attempt in South Korean women.	South Korean adult women (20–39 years old) and adolescent girls (13–18 years old).	975 participantes.
Kreski et al. (2021)	USA	In girls: Small positive association in the general continuous model (β = 0.018), but not significant in stratified analyses, except in the lowest risk stratum.	Social Media Use and Depressive Symptoms Among United States Adolescents	Cross-sectional observational study	To analyze the association between daily social media use and depressive symptoms.	American adolescents in 8th and 10th grade.	74,472 estudiantes.
Nilsen et al. (2023)	Norway	Significant linear increase in physical health problems from 2014 to 2019, more pronounced in girls (β = 0.08 per year) than in boys (β = 0.03).	Trends in physical health complaints among adolescents from 2014 – 2019: Considering screen time, social media use, and physical activity	Longitudinal study with repeated cross-sectional data	To analyze trends in physical health complaints associated with screen time and social media use.	Norwegian adolescents (13–18 years old).	419,934 participantes.
Oksanen et al. (2021)	USA and Spain	2% of young people purchased drugs online (77% used social media like Instagram/Facebook).	Social Media and Access to Drugs Online: A Nationwide Study in the United States and Spain among Adolescents and Young Adults	Cross-sectional	To explore factors associated with online drug purchasing in adolescents and young adults.	Adolescents and young adults aged 15-25 in the US and Spain	2,000 participantes
Parris et al. (2022)	USA	There was a statistically significant indirect effect of bullying perceptions on mental distress through social media rumination (β = 0.05, 95% confidence interval [CI] = [0.01, 0.11]).	Exploring Social Media Rumination: Associations With Bullying, Cyberbullying, and Distress	Cross-sectional with mediation	To explore the mediating role of social media rumination between bullying and psychological distress.	Socioeconomicall y at-risk adolescents in the US	169 estudiantes (73.7% mujeres)
Plackett et al. (2023)	UK	Self-esteem partially mediated the relationship in unadjusted analyses (68% of the effect; indirect β = 0.70, P = 0.02), but this mediation disappeared when adjusting for covariates (indirect β = 0.24, P = 0.22).	The Longitudinal Impact of Social Media Use on UK Adolescents' Mental Health: Longitudinal Observational Study	Longitudinal observational study (cohort)	To assess the longitudinal impact of social media use on mental health.	British adolescents (10–15 years old).	3,228 participantes.
Pravosud et al. (2024)	USA	98% of participants increased their use of social media during lockdown. 54% increased their use of e-cigarettes.	Social Media Exposure and Other Correlates of Increased e-Cigarette Use Among Adolescents During Remote Schooling: Cross-Sectional Study	Cross-sectional observational study	To explore the association between exposure to social media and e-cigarette use.	Californian adolescents (13-18 years old)	85 adolescentes.
Rossi & DeSilva (2020)	USA	Social media can normalize self-harming/suicidal behaviors, presenting	Social Media Applications: A Potential Avenue for Broadcasting Suicide	Case study	To explore the role of social media in	Young adult suicide attempter	1 caso

Author(s)	Country	Findings	Title	Design	Objective	Population	Sample
		them as valid coping strategies.	Attempts and Self-Injurious Behavior		normalizing suicidal behavior.		
Saintila et al. (2024)	Peru	Men are at higher risk of MetS than women (OR = 1.133, *p* = 0.028). Anxiety symptoms significantly increase the risk	Associations between social network addiction, anxiety symptoms, and risk of metabolic syndrome in Peruvian adolescents—a cross-sectional study	Cross	To explore associations between social media addiction, anxiety, and risk of metabolic syndrome.	Adolescents aged 12-18 in Peru	903 participantes
Shensa et al. (2020)	USA	FTF-ES: Associated with a lower risk of depression (OR = 0.57). SM-ES: Associated with a higher risk of depression (OR = 1.20).}	Emotional support from social media and face-to-face relationships: Associations with depression risk among young adults	Cross	To examine associations between emotional support on social media/face-to-face and risk of depression.	Young adults aged 18-30 in the US	2,375 participantes
Sserunkuuma et al. (2023)	Uganda	16.73% of students presented moderate to severe depressive symptoms (PHQ-9 ≥ 10).	Problematic use of the internet, smartphones, and social media among medical students and relationship with depression: An exploratory study	Cross-sectional	To explore the relationship between problematic internet use and depression in medical students.	Medical students in Uganda	269 participantes
Szlyk et al. (2023)	USA	Adolescents with recent suicidal ideation perceive more unhelpful social interactions (pity, judgments) when following depressive content	How do teens with a history of suicidal behavior and self-harm interact with social media?	Mixed methods (cross-sectional)	To analyze social media interactions and their relationship with suicidal behavior in adolescents.	Adolescents aged 15-17 in the US with a history of suicide risk	93 participantes
Tajjamul & Aleem (2022)	Pakistan	33.6% perceived a negative impact on their mental health	Impacts of Social Media on Mental Health of Youth in Karachi, Pakistan: An Exploratory Study	Cross-sectional	To explore the relationship between social media use and mental health in young people.	Youth aged 15-29 in Karachi, Pakistan	119 estudiantes universitarios
Varela et al. (2023)	Chile	The risk of social media addiction is negatively associated with life satisfaction (r = −0.28).	Wellbeing, social media addiction and coping strategies among Chilean adolescents during the pandemic	Analytical cross-sectional study	To analyze the relationship between social media addiction and life satisfaction.	Chilean adolescents (14–19 years old).	1,290 estudiantes.
Victor et al. (2024)	Malaysia	38.8% reported high levels of depression	Social media addiction and depression among adolescents in two Malaysian states	Cross-sectional	To explore the relationship between social media addiction and depression in adolescents.	Adolescents aged 13-21 in Malaysia	384 participantes
Yıldırım et al. (2023)	Turkey	Problematic social media use and sleep problems correlate positively (*r* = 0.36, *p* < 0.01).	Fear of COVID-19 and Sleep Problems in Turkish Young Adults: Mediating Roles of Happiness and Problematic Social Networking Sites Use	Cross-sectional with mediation	To analyze the mediating role of happiness and problematic social media use between fear of COVID-19 and sleep problems.	Young adults in Turkey (18-35 years old)	506 participantes (67,6 % mujeres; edad media = 28,23 ± 8,73 años)
Zani et al. (2024)	Indonesia	Excessive social media use is associated with mental health problems and lower academic performance. Multilevel interventions are recommended to promote emotional regulation and resilience.	The Relationship Between Social Media Dependency, Mental Health, and Academic Performance Among Adolescents in Indonesia	Cross-sectional observational study	To explore the relationship between social media dependence, mental health, and academic performance.	Indonesian adolescents (15–18 years old).	200 estudiantes.

Table S2. Risk of bias of the included studies

Study (Year)	Design	Tool	Risk of Bias	Main sources of bias
Nilsen et al. (2023)	Cross	AXIS	Moderate	Self-report
Oksanen et al. (2021)	Cross	AXIS	Low	NA

Parris et al. (2022)	Cross	AXIS	Moderate	Selective sample
Plackett et al. (2023)	Longitudinal	NOS	Moderate	Self-report
Pravosud et al. (2024)	Cross	AXIS	Moderate	Non-representative sample. Self-report
Saintila et al. (2024)	Cross	AXIS/STROBE	Moderate	Non-probabilistic sample
Shensa et al. (2023)	Cross	AXIS	Moderate	Possible self-selection
Szlyk et al. (2023)	Cross	AXIS/STROBE	Moderate	Non-probabilistic sample
Victor et al. (2024)	Cross	AXIS	Moderate	Non-ramdom sample
Yıldırım et al. (2023)	Cross	AXIS	Moderate	Lack of control of confusing facts
Zani et al. (2024)	Cross	AXIS	Moderate	Limited control of confusing. Non-representative sample
Abrutyn et al. (2023)	Qualitative	CASP	Moderate	Non-representative sample
Rossi (2020)	Qualitative	CASP	High	Limited control of confusing
Balt et al. (2023)	Qualitative	CASP	Moderate	Non-random sample. Limited control of confusing
Ávalos Ruiz et al. (2022)	Cross	AXIS/NOS	Moderate	Non-representative sample. Self-report
Beeres et al. (2021)	Longitudinal	ROBINS-I	Moderate	Differential attrition. Limited control of confusing
Borraccino et al. (2022)	Cross	AXIS/NOS	Low	NA
Farias et al. (2024)	Cross	AXIS	Moderate	Non reprsentive sample. Limited control of confusing.
Hamilton & Lee (2021)	Cross	AXIS/JBI	Moderate	Self-report
Kim et al. (2024)	Cross	AXIS/ROBINS-I	Moderate	Self-report
Kreski et al. (2021)	Cross	AXIS	Moderate	NA
Varela et al. (2023)	Cross	AXIS/NOS	Moderate	
Sserunkuuma et al. (2023)	Cross	AXIS/JBI	Moderate	Non-probabilistic sample
De Felice et al. (2022)	Qualitative	CASP	Low	
Craig et al. (2020)	Cross	AXIS/ROBINS-I	Moderate	
Tajjamul & Aleem (2022)	Cross	AXIS/NOS	Moderate	Non-probabilistic sample
Bucur & Dinu (2020)	Cross	AXIS/ROBINS-I	Moderate	Non-probabilistic sample
Ballesteros-Holmes et al. (2024)	Quasi-experimental	ROBINS-I	Moderate	Non-probabilistic sample
Agbo, M. C. (2021).	Cross	AXIS	High	Limited control of confusing

Table S3. Effect sizes

Author	n	r	OriginalEffect	Design	Disorder	AgeGroup	UseType
Agbo2021-ef1	800	**0,35**	Mean diff.=3.35±0.73 (Likert scale), time on social media - decrease in social activities	Cross	Depression/Anxiety	Adolescents	Use >10h+night
Agbo2021-ef2	800	**0,3**	Mean diff.=3.18±1.00 (Likert scale), nighttime use (2:AM) - insomnia, low mood	Cross	Depression/Anxiety	Adolescents	Use >10h+night
Agbo2021-ef3	800	**0,25**	Mean diff.=2.73±0.74 (Likert scale), comparison with images - low self-esteem	Cross	Depression/Anxiety	Adolescents	Use >10h+night
Agbo2021-ef4	800	**0,4**	Mean diff.=3.25±0.84 (Likert scale), cyberbullying - depressive symptoms	Cross-sectional	Depression/Anxiety	Adolescents	Use >10h+night
Ávalos Ruiz et al.2022-ef1	25	0,43	Video game addiction and depression, r=0.428;<br>r=0.428	Cross-sectional observational study	RRSS Adicction	Adolescents	Technological addiction (video games as a proxy)
Ávalos Ruiz et al.2022-ef2	25	0,46	General technology addiction and bullying;<br>r=0.458	Cross-sectional observational study	RRSS Adicction	Adolescents	Technological addiction (video games as a proxy)
Beeres et al.2021-ef1	3501	0,32	Interpersonal association, B=2.40; B=2.40. Intrapersonal association was not reported;<br>r=0.41 (Weighted average externalizing + internalizing), r=0.36 (externalizing), r=0.32 (internalizing)	Longitudinal	Physical/Mental Health	Adolescents	Frequency of use (hours/week)
Beeres et al.2021-ef2	3501	0,36	Interpersonal association, B=2.40; B=2.40. Intrapersonal association was not reported;<br>r=0.41 (Weighted average externalizing + internalizing), r=0.36 (externalizing), r=0.32 (internalizing)	Longitudinal	Physical/Mental Health	Adolescents	Frequency of use (hours/week)
Borraccino et al.2022-ef1	58976	**0,18**	OR PSMU - Cybervictimization = 2.33	Cross	Digital aggression	Adolescents	Recreational use (between peers)
Borraccino et al.2022-ef2	58976	**0,22**	OR PSMU - Cyberbullying perpetration = 3.18.	Cross	Digital aggression	Adolescents	Recreational use (between peers)
Bucur & Dinu (2020)2020-ef1	820	**0,35**	Weighted average (cybervictimization perpetration) = 0.20"	Cross	Depression/Anxiety	Adol_Young	Linguistic expression (text analysis)
Bucur & Dinu (2020)2020-ef2	820	**0,42**	ERDE50: 6.44%. (1st person's pronoun); r=0.42	Cross	Depression/Anxiety	Adol_Young	Linguistic expression (text analysis)
Bucur & Dinu (2020)2020-ef3	820	**0,28**	FI: 0.38 (Mention of Medical Topics: Self-harm); 0.28	Cross	Depression/Anxiety	Adol_Young	Linguistic expression (text analysis)
Craig et al.2020-ef1	166647	**0,08**	OR: Problematic use, victimization: 1.4	Observational cross-sectional	Digital aggression	Adolescents	Daily use (>4h/day)
Craig et al.2020-ef2	166647	**0,11**	OR: Problematic use, perpetration: 1.54	Observational cross-sectional	Digital aggression	Adolescents	Daily use (>4h/day)
Craig et al.2020-ef3	166647	**0,04**	OR Heavy use, victimization: 1.17	Observational cross-sectional	Digital aggression	Adolescents	Daily use (>4h/day)
Craig et al.2020-ef4	166647	**0,07**	OR Heavy use, perpetration: 1.34	Observational Cross-Sectional	Digital aggression	Adolescents	Daily use (>4h/day)
Craig et al.2020-ef5	166647	**0,06**	OR Contact with strangers, victimization: 1.27	Observational Cross-Sectional	Digital aggression	Adolescents	Daily use (>4h/day)
Craig et al.2020-ef6	166647	**0,07**	OR Contact with strangers, perpetration: 1.37	Observational Cross-Sectional	Digital aggression	Adolescents	Daily use (>4h/day)
Farias et al.2024-ef1	370	0,37	OR Twitter = 10.7	Observational Cross-Sectional Study	Eating disorder	Young	Social comparison (body idealization)
Farias et al.2024-ef2	370	0,20	OR Interest in influencers = 2.41	Observational cross-sectional study	Eating disorder	Young	Social comparison (body idealization)

Author	n	r	OriginalEffect	Design	Disorder	AgeGroup	UseType
Farias et al.2024-ef3	370	0,21	OR High advertising exposure = 2.57	Observational cross-sectional study	Eating disorder	Young	Social comparison (body idealization)
Farias et al.2024-ef4	370	0,18	OR Tik Tok = 2.08	Observational cross-sectional study	Eating disorder	Young	Social comparison (body idealization)
Farias et al.2024-ef5	370	0,35	YouTube OR (>2.5 hours) = 9.04; Weighted social media OR = 3.33; Weighted r = 0.26	Observational cross-sectional study	Eating disorder	Young	Social comparison (body idealization)
Hamilton & Lee.2021-ef1	4153	**0,35**	Posting frequency β = 0.70	Cross-sectional	Sleep disorder	Adolescents	Passive use (lurking)
Hamilton & Lee.2021-ef2	4153	**0,38**	Review frequency β = 0.76	Cross-sectional	Sleep disorder	Adolescents	Passive use (lurking)
Hamilton & Lee.2021-ef3	4153	**0,18**	Importance of social media for engagement B = 0.36	Cross-sectional	Sleep disorder	Adolescents	Passive use (lurking)
Hamilton & Lee.2021-ef4	4153	**0,02**	Interaction: Social Media Frequency x Importance B = 0.04; Avg. Pond (post, query, importance) B = 0.61; Avg. Pond (post, query, importance + interaction) B = 0.47.	Cross-sectional	Sleep disorder	Adolescents	Passive use (lurking)
Kim et al.2024*ef1	975	0,28	Time spent on social media SHAP = 0.25, adolescents; r = 0.23	Analytical cross-sectional study	Suicidal behavior	Adol_Young	Comparative use (idealization of lifestyles)
Kim et al.2024*ef2	975	0,35	Freq. Use for communication, SHAP = 0.18, adolescents; r = 0.18	Analytical cross-sectional study	Suicidal behavior	Adol_Young	Comparative use (idealization of lifestyles)
Kim et al.2024*ef3	975	0,25	Online sexual victimization, SHAP=0.28, Adolescents. r=0.28	Analytical cross-sectional study	Suicidal behavior	Adol_Young	Comparative use (idealization of lifestyles)
Kim et al.2024*ef4	975	0,40	Internet problematic use, SHAP=0.25, Adolescents and young adults; r=0.23	Analytical cross-sectional study	Suicidal behavior	Adol_Young	Comparative use (idealization of lifestyles)
Kim et al.2024*ef6	975	0,21	Motivation, habitual use (for leisure time) SHAP=0.13; r=0.13; Weighted average (SHAP)=0.22. Weighted average r=0.21	Analytical cross-sectional study	Suicidal behavior	Adol_Young	Comparative use (idealization of lifestyles)
Kreski et al.2021-ef1	32000	0,00	Daily social media use (girls), symptoms of depression, B=0.018	Observational cross-sectional study	Depression/Anxiety	Adolescents	Daily use (self-reported frequency)
Kreski et al.2021-ef2	15000	-0,03	Daily social media use (girls), symptoms >10, OR=0.81	Observational cross-sectional study	Depression/Anxiety	Adolescents	Daily use (self-reported frequency)
Kreski et al.2021-ef4	15000	0,07	Daily social media use (boys), symptoms of depression >12, OR=1.42	Observational cross-sectional study	Depression/Anxiety	Adolescents	Daily use (self-reported frequency)
Kreski et al.2021-ef5	8000	-0,03	Daily social media use (boys), symptoms of depression >, OR(low risk)=0.77;	Observational cross-sectional study	Depression/Anxiety	Adolescents	Daily use (self-reported frequency)
Nilsen et al.2023-ef1	402826	0,21	Weighted average r=-0.003;	Longitudinal study with repeated cross-sectional data	Physical/Mental Health	Adolescents	General/Recreational
Nilsen et al.2023-ef2	402826	0,09	Weighted average AUC=0.81	Longitudinal study with repeated cross-sectional data	Physical/Mental Health	Adolescents	General/Recreational
Oksanen et al.2021-ef1	1212	0,07	Online Friends OR = 1.58	Cross	Substance use	Adol_Young	Recreational use (social interaction)
Oksanen et al.2021-ef2	259	0,16	Social Media Bubble OR = 1.25	Cross	Substance use	Adol_Young	Recreational use (social interaction)
Oksanen et al.2021-ef3	2424	0,11	Excessive Internet Use OR = 1.11	Cross	Substance use	Adol_Young	Recreational use (social interaction)
Oksanen et al.2021-ef4	2424	0,05	Social Media Activity (high freq) OR = 1.15	Cross	Substance use	Adol_Young	Recreational use (social interaction)

Author	n	r	OriginalEffect	Design	Disorder	AgeGroup	UseType
Parris et al.2022	169	0,37	r = 0.37, for rumination; Not reported for Social Media Use"	Cross-sectional with mediation	Digital aggression	Adolescents	Social rumination (associated with bullying/cyberbullying)
Plackett et al.2023-ef1	2603	0,03	Active social media use (adjusted), β = 0.21	Longitudinal observational study	RRSS Adicction	Adolescents	Daily use (frequency)
Plackett et al.2023-ef2	2945	0,11	Active social media use (unadjusted), β = 0.96	Longitudinal observational study	RRSS Adicction	Adolescents	Daily use (frequency)
Pravosud et al.2024	63	0,24	AOR = 2.34 (exposure to digital content)	Cross-sectional observational study	Physical/Mental Health	Adolescents	Night use (>2h/night)
Saintila et al.2024	903	0,10	OR social media addiction = 1.517 (variable of interest);	Cross	Eating disorder	Adolescents	Compulsive use (>5h/day)
Shensa et al.2020-ef1	2375	**0,09**	OR anxiety = 2.596	Cross	Depression/Anxiety	Adolescents	Compulsive use (>5h/day)
Shensa et al.2020-ef2	2375	**0,05**	OR hours spent on social media per day = 1.10	Cross	Depression/Anxiety	Adolescents	Compulsive use (>5h/day)
Sserunkuuma et al.2022-ef1	269	0,23	Twitter, β = -1.88 (SE = 0.57); r = -0.23	Cross	RRSS Adicction	Young	Addiction/Dependence
Sserunkuuma et al.2022-ef2	269	0,51	Social media addiction (BSMAS), r = 0.51 (p < 0.001); r = 0.51; Weighted average correlations r = 0.14	Cross-sectional	RRSS Adicction	Young	Addiction/Dependence
Szlyk H.S. et al.2023-ef1	93	0,30	χ2 = 7.1, posting depressive content on social media	Mixed methods (cross-sectional)	Suicidal behavior	Adolescents	Interaction with depressive content
Szlyk H.S. et al.2023-ef2	93	0,28	χ2 = 5.8, Following depressive content	Mixed methods (cross-sectional)	Suicidal behavior	Adolescents	Interaction with depressive content
Szlyk H.S. et al.2023-ef3	93	0,22	χ2 = 4.9. Interaction with peers in crisis (response to peers with suicidal ideation)	Mixed methods (cross-sectional)	Suicidal behavior	Adolescents	Interaction with depressive content
Tajjamul & Aleem.2022-ef1	119	**0,42**	89.1% (106/119), daily frequency of use - increased anxiety	Cross-sectional	Depression/Anxiety	Young	General use (no specification)
Tajjamul & Aleem.2022-ef2	119	**0,18**	33.6% (40/119), perceived negative impact - increased stress levels	Cross-sectional	Depression/Anxiety	Young	General use (no specification)
Tajjamul & Aleem.2022-ef3	119	**0,22**	43.7% (52/119), nighttime use - associated with sleep disturbances	Cross-sectional	Depression/Anxiety	Young	General use (no specification)
Tajjamul & Aleem.2022-ef4	119	**0,15**	38.7% (46/119), preference for private communication - does not reduce feelings of loneliness	Cross-sectional	Depression/Anxiety	Young	General use (no specification)
Tajjamul & Aleem.2022-ef5	119	**0,3**	59.8% (70/117), use among young people - increases frequency of anxiety episodes	Cross-sectional	Depression/Anxiety	Young	General use (no specification)
Varela et al.2023	1290	0,28	β=−0.09; r=−0.28; Sample of 1,290 participants	Analytical cross-sectional study	RRSS Adicction	Adolescents	Social use (connection with peers)
Victor et al.2024-ef1	384	0,21	r=0.206 (social media addiction)	Cross-sectional	RRSS Adicction	Adolescents	Addiction (loss of control)
Victor et al.2024-ef2	384	0,20	n2=0.04 (anova) (Time on social media >7h/day)	Cross-sectional	RRSS Adicction	Adolescents	Addiction (loss of control)
Victor et al.2024-ef3	384	0,29	r=0.294 (number of platforms used)	Cross-sectional	RRSS Adicction	Adolescents	Addiction (loss of control)
Victor et al.2024-ef4	384	0,14	r=0.144 (Daily check frequency)	Cross-sectional	RRSS Adicction	Adolescents	Addiction (loss of control)
Yıldırım et al.2023	506	0,27	Social Media Problems, β=0.27	Cross-sectional with mediation	Sleep disorder	Young	Problematic use (mediator)
Zani et al.2024-ef1	200	0,33	Higher. Social Media Dependence - More. Negative Signs. Mental Health, r=0.65	Observational cross-sectional study	Physical/Mental Health	Adolescents	Dependency (use for evasion)

Author	n	r	OriginalEffect	Design	Disorder	AgeGroup	UseType
Zani et al.2024-ef2	200	0,24	Higher. Social Media Dependence - Less. Academic Performance, r=0.66	Observational cross-sectional study	Physical/Mental Health	Adolescents	Dependency (use for evasion)

Note: *Studies with more than one measure of effect are presented according to the number of disorders assessed*

Figure S1. *Meta-regression: Explained variability (R2 > 87%) and effects on TypeUse categories*

```
Mixed-Effects Model (k = 62; tau^2 estimator: REML)

tau^2 (estimated amount of residual heterogeneity):     0.0022 (SE = 0.0007)
tau (square root of estimated tau^2 value):             0.0467
I^2 (residual heterogeneity / unaccounted variability): 98.14%
H^2 (unaccounted variability / sampling variability):   53.83

Test for Residual Heterogeneity:
QE(df = 40) = 696.6646, p-val < .0001

Test of Moderators (coefficients 1:22):
QM(df = 22) = 959.0790, p-val < .0001

Model Results:

                                                                  estimate      se     zval    pval    ci.lb   ci.ub
TipoUsoAdicción (pérdida de control)                                0.2149  0.0347   6.2001  <.0001   0.1470  0.2829  ***
TipoUsoAdicción tecnológica (videojuegos como proxy)                0.4761  0.1543   3.0850  0.0020   0.1736  0.7786   **
TipoUsoBúsqueda de apoyo emocional (vs. interacción cara a cara)    0.0701  0.0361   1.9442  0.0519  -0.0006  0.1409    .
TipoUsoComparación social (idealización corporal)                   0.2704  0.0313   8.6304  <.0001   0.2090  0.3317  ***
TipoUsoDependencia (uso para evasión)                               0.2966  0.0602   4.9233  <.0001   0.1785  0.4146  ***
TipoUsoExpresión lingüística (análisis de textos)                   0.3669  0.0337  10.8907  <.0001   0.3009  0.4330  ***
TipoUsoFrecuencia de uso (horas/semana)                             0.3543  0.0351  10.0859  <.0001   0.2854  0.4231  ***
TipoUsoInteracción con contenido depresivo                          0.2736  0.0666   4.1105  <.0001   0.1432  0.4041  ***
TipoUsoRumia social (asociada a bullying/cyberbullying)             0.3884  0.0906   4.2879  <.0001   0.2109  0.5660  ***
TipoUsoUso comparativo (idealización de estilos de vida)            0.3091  0.0253  12.1972  <.0001   0.2594  0.3587  ***
TipoUsoUso compulsivo (>5h/día)                                     0.1003  0.0574   1.7485  0.0804  -0.0121  0.2128    .
TipoUsoUso diario (>4h/día)                                         0.0718  0.0191   3.7614  0.0002   0.0344  0.1092  ***
TipoUsoUso diario (frecuencia auto-reportada)                       0.0037  0.0237   0.1575  0.8748  -0.0428  0.0503
TipoUsoUso diario (frecuencia)                                      0.0706  0.0357   1.9791  0.0478   0.0007  0.1405    *
TipoUsoUso excesivo (≥10 h/día + nocturno)                          0.3385  0.0293  11.5491  <.0001   0.2811  0.3960  ***
TipoUsoUso genera                                                   0.2628  0.0465   5.6539  <.0001   0.1717  0.3539  ***
TipoUsoUso nocturno (>2h/noche)                                     0.2448  0.1373   1.7829  0.0746  -0.0243  0.5139    .
TipoUsoUso pasivo (lurking)                                         0.3828  0.0348  10.9974  <.0001   0.3145  0.4510  ***
TipoUsoUso problemático (mediador)                                  0.2769  0.0646   4.2876  <.0001   0.1503  0.4034  ***
TipoUsoUso recreativo (entre pares)                                 0.2028  0.0332   6.1172  <.0001   0.1378  0.2678  ***
TipoUsoUso recreativo (interacción social)                          0.0881  0.0281   3.1366  0.0017   0.0331  0.1432   **
TipoUsoUso social (conexión con pares)                              0.2877  0.0544   5.2889  <.0001   0.1811  0.3943  ***

---
Signif. codes:  0 '***' 0.001 '**' 0.01 '*' 0.05 '.' 0.1 ' ' 1
```

Figure S2. Diagnosis of influence on combined effect

Phase 1

	rstudent	dffits	cook.d	cov.r	tau2.del	QE.del	hat	weight	inf
1	-0.1162	-0.1162	0.0135	2.1515	0.0062	4138.4554	0.5000	0.2282	
2	0.1162	0.1162	0.0135	2.1515	0.0062	4138.4554	0.5000	0.2282	
3	-0.3935	-0.3935	0.1591	3.3137	0.0063	4134.8913	0.5000	1.8309	
4	0.3935	0.3935	0.1591	3.3137	0.0063	4134.8913	0.5000	1.8309	
5	-0.3698	-0.3698	0.1409	3.4246	0.0063	4087.2623	0.5000	1.9108	
6	0.3698	0.3698	0.1409	3.4246	0.0063	4087.2623	0.5000	1.9108	
7	1.4130	0.7065	0.4911	0.8205	0.0060	4132.0752	0.2000	1.3275	
8	-0.8003	-0.4001	0.1611	1.4596	0.0062	4136.3732	0.2000	1.3275	
9	-0.6757	-0.3378	0.1152	1.5810	0.0062	4136.9709	0.2000	1.3275	
10	-1.0499	-0.5249	0.2751	1.1965	0.0061	4134.8884	0.2000	1.3275	
11	1.1314	0.5657	0.3186	1.1091	0.0061	4134.3224	0.2000	1.3275	
12	1.8549	1.0709	1.0626	0.3250	0.0057	4053.9771	0.2500	1.8437	
13	2.4707	1.4264	1.7415	0.0761	0.0052	4000.0086	0.2500	1.8437	
14	-0.8617	-0.4975	0.2496	1.5549	0.0062	4118.6240	0.2500	1.8437	
15	-3.9270	-2.2673	3.4270	0.0009	0.0040	3866.0864	0.2500	1.8437	*
16	-0.2790	-0.1395	0.0199	2.0509	0.0063	4137.9148	0.2000	1.6413	
17	0.7398	0.3699	0.1384	1.5914	0.0062	4134.6097	0.2000	1.6413	
18	-0.7037	-0.3519	0.1254	1.6365	0.0062	4134.9724	0.2000	1.6413	
19	1.5383	0.7692	0.5718	0.6141	0.0059	4122.5203	0.2000	1.6413	
20	-1.2759	-0.6379	0.4006	0.8988	0.0060	4127.2965	0.2000	1.6413	
21	0.0058	0.0034	0.0000	2.4869	0.0064	4137.9425	0.2517	1.9063	
22	-0.4861	-0.2808	0.0809	2.1355	0.0063	4112.3370	0.2502	1.8955	
23	0.9736	0.5625	0.3169	1.3783	0.0062	4062.4233	0.2502	1.8955	
24	-0.4832	-0.2774	0.0790	2.1285	0.0063	4126.1088	0.2479	1.8778	
25	1.1131	1.1131	1.2288	1.7259	0.0061	1094.9242	0.5000	1.9153	
26	-1.1131	-1.1131	1.2288	1.7259	0.0061	1094.9242	0.5000	1.9153	
27	-0.3071	-0.1853	0.0352	2.2677	0.0063	4138.2736	0.2618	1.6888	
28	0.7589	0.3576	0.1287	1.4597	0.0062	4136.7719	0.1816	1.1715	
29	0.2582	0.1575	0.0255	2.3085	0.0063	4135.2001	0.2783	1.7954	
30	-0.6094	-0.3796	0.1470	2.0337	0.0063	4134.5584	0.2783	1.7954	
31	NA	NA	NA	NA	NA	NA	1.0000	0.9676	
32	-0.6995	-0.6971	0.4937	2.6916	0.0063	4129.5403	0.4983	1.8032	
33	0.6995	0.7019	0.5005	2.7099	0.0063	4129.5403	0.5017	1.8156	
34	NA	NA	NA	NA	NA	NA	1.0000	0.5162	
35	NA	NA	NA	NA	NA	NA	1.0000	1.6227	
36	0.3461	0.3461	0.1231	3.3487	0.0063	4136.5539	0.5000	1.7930	
37	-0.3461	-0.3461	0.1231	3.3487	0.0063	4136.5539	0.5000	1.7930	
38	-2.4038	-2.4038	5.4485	0.3614	0.0056	4124.1149	0.5000	1.1888	*
39	2.4038	2.4038	5.4485	0.3614	0.0056	4124.1149	0.5000	1.1888	*
40	0.3340	0.2362	0.0560	1.8282	0.0062	4138.2967	0.3333	0.6824	
41	0.1308	0.0925	0.0086	1.8675	0.0062	4138.4440	0.3333	0.6824	
42	-0.4650	-0.3288	0.1085	1.7857	0.0062	4138.1337	0.3333	0.6824	
43	NA	NA	NA	NA	NA	NA	1.0000	1.7010	
44	-0.0725	-0.0418	0.0018	2.0659	0.0063	4138.4529	0.2500	1.3427	
45	-0.1490	-0.0860	0.0075	2.0504	0.0063	4138.3953	0.2500	1.3427	
46	1.0869	0.6275	0.3926	1.2327	0.0061	4134.5348	0.2500	1.3427	
47	-0.8601	-0.4966	0.2477	1.4933	0.0062	4135.9879	0.2500	1.3427	
48	NA	NA	NA	NA	NA	NA	1.0000	1.4477	
49	0.6755	0.6755	0.4589	2.4082	0.0062	4137.4562	0.5000	1.0494	
50	-0.6755	-0.6755	0.4589	2.4082	0.0062	4137.4562	0.5000	1.0494	
51	0.1146	0.0513	0.0027	2.2235	0.0064	4124.5354	0.1667	1.9142	
52	0.5328	0.2383	0.0582	1.8750	0.0063	3840.1579	0.1667	1.9142	
53	-0.4380	-0.1959	0.0395	1.9866	0.0063	3936.2227	0.1667	1.9142	
54	-0.0235	-0.0105	0.0001	2.2412	0.0064	4137.8868	0.1667	1.9142	
55	-0.1614	-0.0722	0.0054	2.2054	0.0064	4110.8556	0.1667	1.9142	
56	-0.0235	-0.0105	0.0001	2.2412	0.0064	4137.8868	0.1667	1.9142	
57	1.7107	0.8553	0.7235	0.7705	0.0060	4133.5138	0.2000	0.7975	
58	-0.7425	-0.3713	0.1382	1.4040	0.0062	4137.5237	0.2000	0.7975	
59	-0.3592	-0.1796	0.0324	1.5684	0.0062	4138.2486	0.2000	0.7975	
60	-1.0275	-0.5137	0.2638	1.2322	0.0061	4136.6629	0.2000	0.7975	
61	0.4288	0.2144	0.0462	1.5459	0.0062	4138.1542	0.2000	0.7975	
62	0.3578	0.2066	0.0436	2.0988	0.0063	4137.6996	0.2500	1.5912	
63	-0.3851	-0.2223	0.0505	2.0765	0.0063	4137.5778	0.2500	1.5912	
64	-1.1187	-0.6459	0.4146	1.1723	0.0061	4131.1335	0.2500	1.5912	
65	1.1471	0.6623	0.4353	1.1344	0.0061	4130.7672	0.2500	1.5912	
66	-0.0211	-0.0149	0.0002	2.5311	0.0063	4138.4680	0.3333	1.5978	
67	1.1567	0.8179	0.6636	1.2610	0.0061	4130.4792	0.3333	1.5978	
68	-1.1346	-0.8023	0.6392	1.2941	0.0061	4130.7725	0.3333	1.5978	

Phase 2

	rstudent	dffits	cook.d	cov.r	tau2.del	QE.del	hat	weight	inf
1	-0.1193	-0.1193	0.0142	2.0963	0.0035	3851.7152	0.5000	0.1581	
2	0.1193	0.1193	0.0142	2.0963	0.0035	3851.7152	0.5000	0.1581	
3	-0.5127	-0.5127	0.2703	3.2026	0.0036	3848.1511	0.5000	2.0441	
4	0.5127	0.5127	0.2703	3.2026	0.0036	3848.1511	0.5000	2.0441	
5	-0.4887	-0.4887	0.2469	3.3737	0.0036	3800.5221	0.5000	2.2004	
6	0.4887	0.4887	0.2469	3.3737	0.0036	3800.5221	0.5000	2.2004	
7	1.6942	0.8471	0.6999	0.6232	0.0033	3845.3351	0.2000	1.2432	
8	-0.9576	-0.4788	0.2295	1.2904	0.0035	3849.6330	0.2000	1.2432	
9	-0.8083	-0.4042	0.1641	1.4289	0.0035	3850.2307	0.2000	1.2432	
10	-1.2572	-0.6286	0.3920	1.0024	0.0035	3848.1482	0.2000	1.2432	
11	1.3552	0.6776	0.4540	0.9106	0.0034	3847.5822	0.2000	1.2432	
12	0.9932	0.7023	0.4935	1.5128	0.0035	3836.4067	0.3333	2.0686	
13	1.7539	1.2402	1.4225	0.4110	0.0032	3807.5651	0.3333	2.0686	
14	-3.1286	-2.2122	3.5933	0.0097	0.0025	3740.2112	0.3333	2.0686	*
15	-0.3512	-0.1756	0.0316	1.9995	0.0036	3851.1746	0.2000	1.7088	
16	0.9350	0.4675	0.2193	1.3360	0.0035	3847.8695	0.2000	1.7088	
17	-0.8890	-0.4445	0.1987	1.3968	0.0035	3848.2322	0.2000	1.7088	
18	1.9736	0.9868	0.9060	0.2903	0.0032	3835.7801	0.2000	1.7088	
19	-1.6270	-0.8135	0.6347	0.5357	0.0033	3840.5563	0.2000	1.7088	
20	0.0066	0.0040	0.0000	2.6509	0.0037	3851.2023	0.2529	2.1915	
21	-0.6431	-0.3715	0.1415	1.9779	0.0036	3825.5968	0.2504	2.1697	
22	1.3040	0.7536	0.5515	0.8395	0.0034	3775.6831	0.2504	2.1697	
23	-0.6363	-0.3638	0.1357	1.9765	0.0036	3839.3686	0.2463	2.1348	
24	1.5103	1.5103	2.1572	0.8422	0.0033	808.1840	0.5000	2.2095	*
25	-1.5103	-1.5103	2.1572	0.8422	0.0033	808.1840	0.5000	2.2095	*
26	-0.3510	-0.2140	0.0471	2.2988	0.0036	3851.5334	0.2634	1.7884	
27	0.8999	0.3839	0.1477	1.2704	0.0035	3850.0317	0.1539	1.0449	
28	0.3795	0.2390	0.0586	2.2329	0.0036	3848.4599	0.2913	1.9776	
29	-0.7546	-0.4852	0.2397	1.9193	0.0036	3847.8182	0.2913	1.9776	
30	NA	NA	NA	NA	NA	NA	1.0000	0.8125	
31	-0.9125	-0.9072	0.8280	2.2064	0.0035	3842.8001	0.4971	1.9921	
32	0.9125	0.9177	0.8474	2.2319	0.0035	3842.8001	0.5029	2.0153	
33	NA	NA	NA	NA	NA	NA	1.0000	0.3837	
34	NA	NA	NA	NA	NA	NA	1.0000	1.6783	
35	0.4476	0.4476	0.2061	3.2768	0.0036	3849.8137	0.5000	1.9733	
36	-0.4476	-0.4476	0.2061	3.2768	0.0036	3849.8137	0.5000	1.9733	
37	0.3633	0.2569	0.0662	1.7311	0.0035	3851.5565	0.3333	0.5296	
38	0.1423	0.1006	0.0102	1.7637	0.0035	3851.7038	0.3333	0.5296	
39	-0.5058	-0.3576	0.1281	1.6958	0.0035	3851.3935	0.3333	0.5296	
40	NA	NA	NA	NA	NA	NA	1.0000	1.8094	
41	-0.0868	-0.0501	0.0026	1.9768	0.0036	3851.7127	0.2500	1.2635	
42	-0.1785	-0.1031	0.0108	1.9575	0.0036	3851.6551	0.2500	1.2635	
43	1.3052	0.7535	0.5622	1.0165	0.0034	3847.7946	0.2500	1.2635	
44	-1.0320	-0.5958	0.3547	1.2999	0.0035	3849.2478	0.2500	1.2635	
45	NA	NA	NA	NA	NA	NA	1.0000	1.4101	
46	0.7738	0.7738	0.6005	2.2378	0.0035	3850.7160	0.5000	0.9023	
47	-0.7738	-0.7738	0.6005	2.2378	0.0035	3850.7160	0.5000	0.9023	
48	0.1511	0.0676	0.0048	2.3566	0.0037	3837.7952	0.1667	2.2072	
49	0.7067	0.3161	0.1021	1.6924	0.0036	3553.4177	0.1667	2.2072	
50	-0.5798	-0.2593	0.0692	1.8949	0.0036	3649.4825	0.1667	2.2072	
51	-0.0309	-0.0138	0.0002	2.3929	0.0037	3851.1466	0.1667	2.2072	
52	-0.2129	-0.0952	0.0095	2.3200	0.0037	3824.1154	0.1667	2.2072	
53	-0.0309	-0.0138	0.0002	2.3929	0.0037	3851.1466	0.1667	2.2072	
54	1.8861	0.9430	0.8814	0.7666	0.0034	3846.7736	0.2000	0.6385	
55	-0.8202	-0.4101	0.1684	1.3336	0.0035	3850.7835	0.2000	0.6385	
56	-0.3969	-0.1984	0.0395	1.4783	0.0035	3851.5084	0.2000	0.6385	
57	-1.1345	-0.5673	0.3214	1.1814	0.0035	3849.9228	0.2000	0.6385	
58	0.4738	0.2369	0.0563	1.4585	0.0035	3851.4140	0.2000	0.6385	
59	0.4468	0.2579	0.0678	2.0051	0.0036	3850.9594	0.2500	1.6276	
60	-0.4809	-0.2777	0.0785	1.9726	0.0036	3850.8377	0.2500	1.6276	
61	-1.4075	-0.8126	0.6454	0.8205	0.0034	3844.3933	0.2500	1.6276	
62	1.4439	0.8336	0.6776	0.7801	0.0034	3844.0270	0.2500	1.6276	
63	-0.0263	-0.0186	0.0004	2.5091	0.0036	3851.7278	0.3333	1.6382	
64	1.4582	1.0311	1.0353	0.8570	0.0034	3843.7390	0.3333	1.6382	
65	-1.4298	-1.0111	0.9973	0.8920	0.0034	3844.0323	0.3333	1.6382	

Phase 3

	rstudent	dffits	cook.d	cov.r	tau2.del	QE.del	hat	weight	dfbs	inf
1	0.9644	0.0687	0.0047	1.0046	0.0169	5039.9994	0.0050	0.5033	0.0688	
2	1.1146	0.0800	0.0064	1.0030	0.0168	5039.3613	0.0050	0.5033	0.0800	
3	0.8798	0.1191	0.0142	1.0210	0.0169	4834.1510	0.0183	1.8289	0.1191	
4	1.2380	0.1738	0.0298	1.0072	0.0167	4749.5274	0.0183	1.8289	0.1737	
5	-0.2731	-0.0442	0.0020	1.0360	0.0172	4491.8715	0.0186	1.8581	-0.0442	
6	0.0477	-0.0004	0.0000	1.0367	0.0172	3897.4238	0.0186	1.8581	-0.0004	
7	1.2388	0.1620	0.0260	1.0062	0.0167	5009.7959	0.0160	1.6012	0.1621	
8	-0.1052	-0.0195	0.0004	1.0318	0.0171	5038.1454	0.0160	1.6012	-0.0195	
9	-0.0306	-0.0100	0.0001	1.0318	0.0171	5037.2233	0.0160	1.6012	-0.0100	
10	-0.2536	-0.0382	0.0015	1.0312	0.0171	5039.7407	0.0160	1.6012	-0.0382	
11	1.0690	0.1379	0.0190	1.0124	0.0168	5014.6768	0.0160	1.6012	0.1379	
12	1.1482	0.1601	0.0254	1.0111	0.0167	4721.6577	0.0183	1.8337	0.1601	
13	1.4271	0.2038	0.0406	0.9983	0.0165	4636.5837	0.0183	1.8337	0.2036	
14	0.5269	0.0659	0.0044	1.0291	0.0171	5004.0817	0.0175	1.7530	0.0659	
15	1.1211	0.1523	0.0230	1.0116	0.0167	4967.9358	0.0175	1.7530	0.1523	
16	0.2842	0.0320	0.0010	1.0329	0.0171	5015.6274	0.0175	1.7530	0.0320	
17	1.5798	0.2225	0.0481	0.9908	0.0164	4933.1819	0.0175	1.7530	0.2224	
18	-0.0320	-0.0109	0.0001	1.0348	0.0172	5027.6795	0.0175	1.7530	-0.0109	
19	-1.6816	-0.2202	0.0470	0.9904	0.0163	4813.2214	0.0186	1.8565	-0.2198	
20	-1.9651	-0.2522	0.0605	0.9740	0.0160	4832.8798	0.0185	1.8526	-0.2516	
21	-1.1441	-0.1559	0.0242	1.0154	0.0168	5038.3448	0.0185	1.8526	-0.1558	
22	-1.9610	-0.2513	0.0601	0.9743	0.0160	4931.5971	0.0185	1.8462	-0.2507	
23	-1.1181	-0.1493	0.0222	1.0157	0.0168	5042.6364	0.0177	1.7730	-0.1493	
24	-0.3896	-0.0534	0.0029	1.0284	0.0171	5041.6156	0.0151	1.5101	-0.0534	
25	-0.8180	-0.1140	0.0131	1.0256	0.0170	5041.7465	0.0182	1.8155	-0.1140	
26	-1.2904	-0.1719	0.0293	1.0093	0.0167	5039.5788	0.0182	1.8155	-0.1718	
27	1.1416	0.1367	0.0186	1.0083	0.0168	5027.9881	0.0137	1.3704	0.1367	
28	-1.4514	-0.1911	0.0359	1.0022	0.0166	5034.3541	0.0182	1.8185	-0.1910	
29	-0.8198	-0.1145	0.0132	1.0257	0.0170	5041.4743	0.0182	1.8232	-0.1145	
30	0.1499	0.0112	0.0001	1.0181	0.0170	5041.5266	0.0094	0.9354	0.0111	
31	-0.8783	-0.1191	0.0143	1.0231	0.0170	5042.8630	0.0174	1.7449	-0.1191	
32	-0.9744	-0.1335	0.0179	1.0210	0.0169	5042.9920	0.0181	1.8146	-0.1335	
33	-1.2900	-0.1718	0.0292	1.0093	0.0167	5039.6483	0.0181	1.8146	-0.1717	
34	0.5528	0.0563	0.0032	1.0181	0.0170	5038.5771	0.0112	1.1212	0.0562	
35	0.4215	0.0417	0.0017	1.0197	0.0170	5039.4064	0.0112	1.1212	0.0416	
36	0.0374	-0.0003	0.0000	1.0221	0.0171	5041.3430	0.0112	1.1212	-0.0003	
37	0.5307	0.0669	0.0045	1.0294	0.0171	4991.4535	0.0178	1.7780	0.0669	
38	-0.0606	-0.0139	0.0002	1.0320	0.0172	5037.3959	0.0161	1.6094	-0.0139	
39	-0.1055	-0.0196	0.0004	1.0319	0.0171	5037.9598	0.0161	1.6094	-0.0196	
40	0.6153	0.0753	0.0057	1.0250	0.0170	5025.3421	0.0161	1.6094	0.0753	
41	-0.5200	-0.0713	0.0051	1.0286	0.0171	5041.7539	0.0161	1.6094	-0.0713	
42	0.4340	0.0516	0.0027	1.0292	0.0171	5024.9908	0.0166	1.6638	0.0516	
43	0.8832	0.1057	0.0112	1.0162	0.0169	5029.7213	0.0143	1.4296	0.1057	
44	0.1921	0.0180	0.0003	1.0275	0.0171	5038.0773	0.0143	1.4296	0.0180	
45	-1.0664	-0.1465	0.0215	1.0183	0.0169	5032.2626	0.0186	1.8593	-0.1465	
46	-0.8280	-0.1167	0.0137	1.0260	0.0170	4942.8527	0.0186	1.8593	-0.1167	
47	-1.3879	-0.1857	0.0340	1.0052	0.0166	4606.3237	0.0186	1.8593	-0.1856	
48	-1.1463	-0.1564	0.0244	1.0154	0.0168	4983.8538	0.0186	1.8593	-0.1564	
49	-1.2265	-0.1662	0.0274	1.0122	0.0167	4896.6435	0.0186	1.8593	-0.1661	
50	-1.1463	-0.1564	0.0244	1.0154	0.0168	4983.8538	0.0186	1.8593	-0.1564	
51	1.4606	0.1685	0.0281	0.9975	0.0166	5027.9689	0.0123	1.2307	0.1688	
52	-0.2224	-0.0295	0.0009	1.0241	0.0171	5041.9756	0.0123	1.2307	-0.0295	
53	0.0391	-0.0003	0.0000	1.0243	0.0171	5040.8619	0.0123	1.2307	-0.0003	
54	-0.4161	-0.0507	0.0026	1.0229	0.0171	5042.5403	0.0123	1.2307	-0.0507	
55	0.5793	0.0620	0.0039	1.0195	0.0170	5037.2969	0.0123	1.2307	0.0619	
56	1.1137	0.1502	0.0224	1.0117	0.0168	4981.4606	0.0173	1.7311	0.1502	
57	0.6878	0.0883	0.0079	1.0252	0.0170	5003.7465	0.0173	1.7311	0.0883	
58	0.2825	0.0316	0.0010	1.0325	0.0171	5020.5603	0.0173	1.7311	0.0316	
59	1.5687	0.2192	0.0468	0.9915	0.0164	4952.9679	0.0173	1.7311	0.2191	
60	1.1147	0.1505	0.0225	1.0117	0.0168	4979.9151	0.0173	1.7341	0.1505	
61	1.7631	0.2499	0.0601	0.9811	0.0162	4937.0251	0.0173	1.7341	0.2497	
62	0.5241	0.0652	0.0043	1.0288	0.0171	5010.2932	0.0173	1.7341	0.0652	
